# When Neuroscience ‘Touches’ Architecture: From Hapticity to a Supramodal Functioning of the Human Brain

**DOI:** 10.3389/fpsyg.2016.00866

**Published:** 2016-06-09

**Authors:** Paolo Papale, Leonardo Chiesi, Alessandra C. Rampinini, Pietro Pietrini, Emiliano Ricciardi

**Affiliations:** ^1^Department of Engineering and Architecture, University of Trieste, TriesteItaly; ^2^Citylab – Laboratory of Social Research on Design, Architecture and Beyond, Department of Political and Social Sciences, School of Architecture, University of Florence, FlorenceItaly; ^3^Department of Surgical, Medical, Molecular Pathology and Critical Area, University of Pisa, PisaItaly; ^4^IMT School for Advanced Studies Lucca, LuccaItaly

**Keywords:** neuroscience, architecture and design, sensory perception, vision, touch, hapticity, supramodality, review

## Abstract

In the last decades, the rapid growth of functional brain imaging methodologies allowed cognitive neuroscience to address open questions in philosophy and social sciences. At the same time, novel insights from cognitive neuroscience research have begun to influence various disciplines, leading to a turn to cognition and emotion in the fields of planning and architectural design. Since 2003, the Academy of Neuroscience for Architecture has been supporting ‘neuro-architecture’ as a way to connect neuroscience and the study of behavioral responses to the built environment. Among the many topics related to multisensory perceptual integration and embodiment, the concept of hapticity was recently introduced, suggesting a pivotal role of tactile perception and haptic imagery in architectural appraisal. Arguments have thus risen in favor of the existence of shared cognitive foundations between hapticity and the supramodal functional architecture of the human brain. Precisely, supramodality refers to the functional feature of defined brain regions to process and represent specific information content in a more abstract way, independently of the sensory modality conveying such information to the brain. Here, we highlight some commonalities and differences between the concepts of hapticity and supramodality according to the distinctive perspectives of architecture and cognitive neuroscience. This comparison and connection between these two different approaches may lead to novel observations in regard to people–environment relationships, and even provide empirical foundations for a renewed evidence-based design theory.

In recent years, novel methodologies to explore the neurobiological bases of mind and behavior have inspired the fields of architecture (e.g., Mallgrave, 2011), planning and urban studies (Portugali, 2004, 2011; van der Veen, 2012; de Lange, 2013), geography (Anderson and Smith, 2001), social sciences and the humanities (Leys, 2002) to open toward cognitive neuroscience and, more specifically, to brain imaging. Novel interdisciplinary fields with the ‘neuro-’ prefix have thus recently emerged, such as *neuro*-economy, *neuro*-law, *neuro*-marketing, and even *neuro*-architecture. A neuroscientific approach to the most diverse fields has proven to be able to offer experimental-based pieces of evidence to different domains, often confirming, reviewing or integrating previous theoretical notions. Yet, when promoting any dialog among disciplines, caution must be urged against certain conceptual ambiguities, as we shall see in this commentary.

## Neuroscience and Architecture

In architecture, new awareness of the complexity of cognitive and emotional processes involved in the daily experience of designed environments has rapidly grown. Such interest also led to the foundation of the Academy of Neuroscience for Architecture (ANFA) in 2003 in San Diego. Since then, various important contributions have emerged from both fields (Eberhard, 2008; Mallgrave, 2011; Robinson and Pallasmaa, 2015).

Provocatively, we may argue that neurophysiology and design started influencing one another during the Renaissance, when anatomists and designers shared their education, studies and the same cultural *milieu*: while Vesalius, Descartes and Willis explored the functional and structural characteristics of the central nervous system, laying the grounds for the subsequent scientific revolution, artists such as Leonardo Da Vinci and Andrea Mantegna spent their days in anatomical observations, visionary hydraulic projects, painting and architectural design.

Since then, design studies and life sciences have been continuously inspiring each other, but only recently have they started to truly share interdisciplinary theoretical and methodological perspectives. Nowadays, the contribution of neuroscientists is actively influencing the architectural debate. For instance, Albright (2015) is approaching design with a neuroscientific perspective on perception and aesthetics. Suggestions on the role of *embodied cognition* through the mirror neuron system in aesthetic response (Freedberg and Gallese, 2007) are taken into account in architectural essays (Mallgrave, 2012; Pallasmaa, 2012; Robinson and Pallasmaa, 2015), and Zeki’s neuroaesthetic theories are being discussed within the architectural field (Mallgrave, 2011). Arbib (2012, 2015) is directly addressing designers with suggestions on sensory perception that could have an impact on design practice.

A specific topic now emerging in the neuro-architectural debate deals with the relationship between sensory experience and architectural perception. The role of non-visual perceptual modalities, and specifically of touch, is currently arousing great interest (e.g., Pallasmaa, 2005). Here, we specifically focus on how the recent neuroscientific evidence of a modality-independent processing of sensory information could actually lead to a ‘sensory intensification’ (i.e., visual and non-visual appreciation of designed spaces) in architectural design.

## Sensory Intensification in Architectural Theory: The Concept Of Hapticity

In the past, many architectural theorists already speculated about the body-architecture relationship, usually in formal theories lacking any experiential or perceptual bases, as in the famous cases of the ‘golden-ratio’ (Markowsky, 1992; Höge, 1995; Falbo, 2005) or other ‘natural’ formal principles, such as those inspired by the supposed preference for natural, living forms (the so-called ‘biophilia hypothesis’ – for a critical assessment see Joye and De Block, 2011).

**FIGURE 1:**
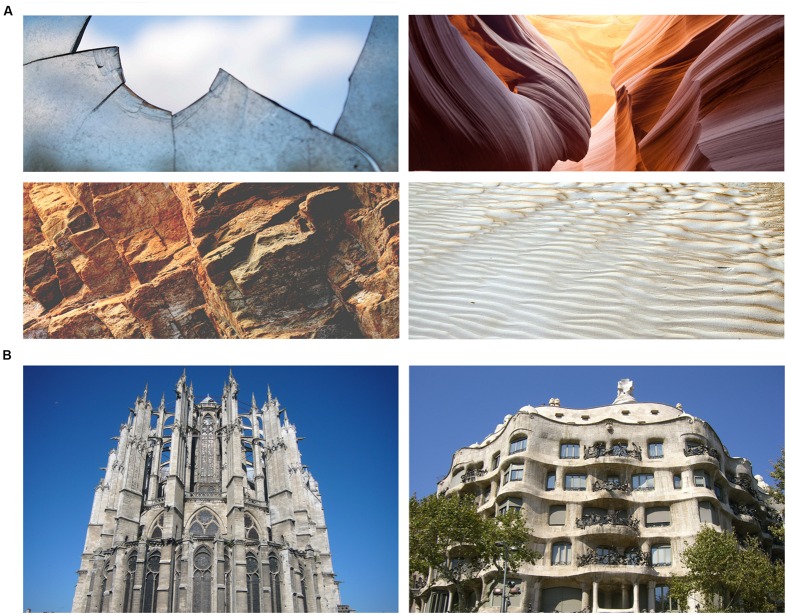
**(A)** According to the notion of *hapticity*, visual cues (e.g., textures or shapes) are able to convey tactile information (e.g., roughness or consistency). Left, top and bottom: edgy shape and texture. Right, top and bottom: smooth shape and texture. Of note, neuroscientific observations showed that the same perceptual information is often processed in a *supramodal* manner, i.e., independently of the modality through which that sensory content is acquired. **(B)** What are the implications of supramodal processing when perceiving architecture, such as the facades of the Beauvais Cathedral (Beauvais, France – on the left) or of the Casa Milà (Barcelona, Spain – on the right)? Has visual appreciation of architecture any non-visual (e.g., tactile) implications as well?

The phenomenological philosophy of Maurice Merleau-Ponty (1964) initiated a theory postulating the embodiment of the built environment into our daily sensorial experience. Similarly, the Danish architect Steen Eiler Rasmussen (1964) favored the importance of perceiving and appreciating architectural features through different sensory modalities, such as in the subtle haptic cues mediated by visual perception: for instance, visual cues on textures and shapes are also able to convey haptic information, as roughness, smoothness or weight, and thus to gratify the eye through sensorimotor imagery (**Figure 1A**). Other authors supported an even tighter relationship between architectural design and embodied cognition, as well as architectural experience and bodily self-consciousness (Mallgrave, 2011; Pasqualini et al., 2013). For instance, the architect Yudell claimed that the visual rhythm of the urban landscape could actually affect body motion (e.g., our walking pace) and excite our imagination toward an enhanced interaction with environmental elements, as in fantasizing about climbing non-existent steps when looking at the unusually textured facade of a skyscraper (in: Bloomer and Moore, 1977).

Currently, multisensory perceptual integration and the role of the sense of touch in architectural design are being explored through the notion of *hapticity*. The term *hapticity* is commonly defined as “the sensory integration of bodily percepts” (Pallasmaa, 2005, 2000) and it suggests a pivotal role of tactile-based (i.e., generally non-visually based) perception and imagery in the architectural experience. The Finnish architect and theorist Pallasmaa hypothesizes the existence of an “unconscious tactile ingredient in vision” (Pallasmaa, 2005) that would be fundamental in architectural appreciation and would exalt touch as the primordial sensory modality.

In this view, even though touch and vision remain intrinsically interwoven in object form and spatial perception, tactile sensations would constitute the core of architectural appraisal (**Figure 1B**). In this sense, for example, it is common to refer to a comfortable and relaxing space as a ‘warm’ place. In this regard, Pallasmaa just recently stressed the importance of sensory experience and our ability to catch complex atmospheres and moods “through simultaneous multi-sensory sensing” (Pallasmaa, 2012). The anthropologist Hall (1966) also emphasized the lack of appeal among designers for the role of haptic sensations, even when visually presented, in bonding people with their environment. Similarly, the architect Sara Robinson (2015) recently reconsidered the privileged link between haptic sensations and emotion.

Consistently, theorists in the architectural field recently advised against the overemphasis on vision as the primary source of aesthetic appreciation, which may result in biased design methodology (O’Neill, 2001; Mallgrave, 2011). Similarly, the neuro-architectural framework claims that the lack of expertise on multi-sensorial appreciation represents a serious limitation in the current design methodology and struggles for a “sensory intensification” in architectural design (Van Kreij, 2008). On the contrary, most practicing architects typically rely on visual representations both during the design process (e.g., sketches and technical drawings) and the subsequent phase of project communication to the public or the client (e.g., 3D models and renders). Moreover, architects rely almost solely on pictures and drawings (in architectural magazines or books) to establish their personal aesthetics and design method (Wastiels et al., 2013).

## Non-Visual Perception and Supramodality in the Human Brain

Visual information plays a crucial role in shaping the manner in which we represent and interact with the world around us. In fact, for sighted people, vision is so pervasive that they find it hard to imagine a world that does not reach them through their eyes. Thanks to the omnipresence of such kind of perceptual information, sighted people tend to think of themselves as ‘visual beings.’ Through preferred metaphors, languages often suggest the dominance of vision over other modalities to construct conceptual knowledge. In English, for example, *knowing* and *seeing* are often used interchangeably in daily conversation, with expressions such as ‘*I see what you mean*,’ ‘*can you see my point?*’ or ‘*seeing is believing.*’ In ancient Greek, the verb root ‘to know’ was used as the past tense of the verb root ‘to see,’ which lacked its own past tense, so that “I saw” was the equivalent of “I knew.”

Consequently, the great majority of psychophysical and neuroscientific studies have been historically focused on the characterization of visual perception and on the dissection of the different steps of visual information processing (e.g., Firestein, 2012) and only recently has non-visual perception started to attract some attention (e.g., Klatzky and Lederman, 2011; Ricciardi and Pietrini, 2011; Ricciardi et al., 2014a; Lacey and Sathian, 2015).

In particular, although vision offers distinctive and unique pieces of information (e.g., colors, perspective, shadows, etc.), several observations indicate that vision might not be so necessary to form a proficient mental representation of the world around us. Indeed, individuals who are visually deprived since birth show perceptual, cognitive, and social skills comparable to those found in sighted individuals (Ricciardi et al., 2006, 2009, 2014a,b; Cattaneo et al., 2008; Pietrini et al., 2009; Ricciardi and Pietrini, 2011; Handjaras et al., 2012, 2016; Heimler et al., 2015). Chris Downey is an architect, Esref Armagan is a painter, Peter Eckert is a photographer: all of them are blind people and yet perfectly capable of successfully conducting their professional lives.

In recent years, functional brain imaging allowed neuroscientists to look at the brains of visually deprived individuals *in vivo* to explore the effects of lack of vision on the formation of proper mental representations. Notably, the question of the extent to which vision is really necessary for the human brain to function, and thus to represent the surrounding world, has recently extended its reach toward a few architectural theorists (Robinson and Pallasmaa, 2015).

Most neuroscientific studies conducted on blind individuals have primarily focused on the structural and functional compensatory plastic rearrangements occurring as a consequence of sensory loss. In sight-deprived individuals, the ‘unisensory’ visual occipital cortex structurally rewires to accommodate non-visual sensory inputs (e.g., Cecchetti et al., 2015), while showing functional cross-modal responses to several non-visual perceptual and cognitive tasks (e.g., Amedi et al., 2005; Frasnelli et al., 2011; Heimler et al., 2014). The loss of a specific sensory modality, such as vision, represents a unique opportunity to understand the real extent to which the brain morphological and functional architecture is programmed to develop independently of any visual experience. Neuroimaging protocols have been suggesting that distinct perceptual tasks evoke comparable patterns of brain responses between congenitally blind and sighted individuals: for instance, both groups show overlapping responses in the ventral temporo-occipital cortex when visually or non-visually recognizing object forms, in the middle temporal area when discriminating motion across sensory modalities and in the dorsal occipito-parietal region when processing spatial information and spatial representations (Amedi et al., 2001, 2002; Pietrini et al., 2004; Ricciardi et al., 2007; Bonino et al., 2008, 2015; for a review: Cattaneo and Vecchi, 2008; Cattaneo et al., 2008; Ricciardi and Pietrini, 2011; Handjaras et al., 2012, 2016; Heimler et al., 2014; Ricciardi et al., 2014a,b).

**FIGURE 2:**
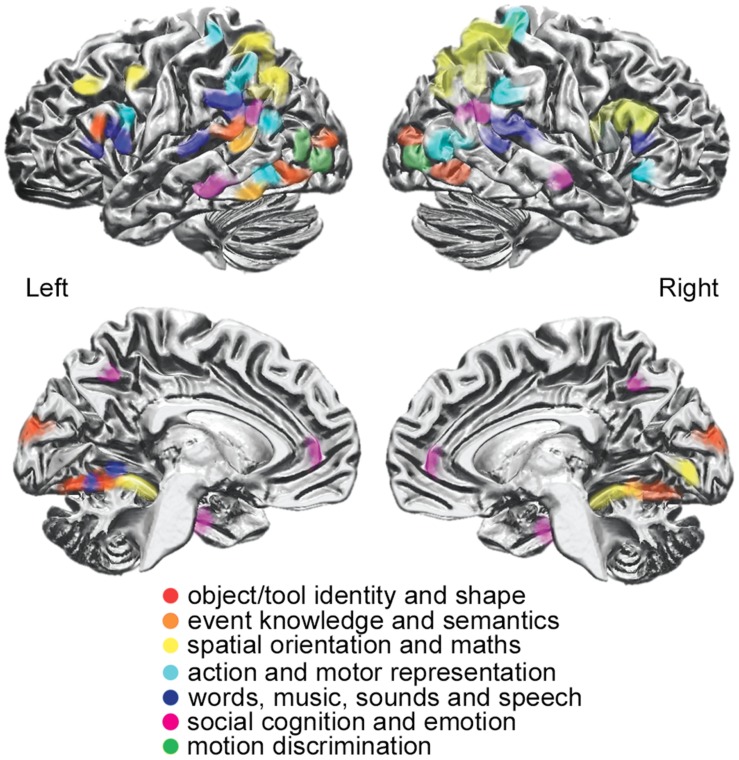
**Supramodal areas showing functional responses to different perceptual, cognitive, and affective stimuli (as shown through different colors), independently from the sensory modality that conveys the information to the brain [modified from Ricciardi et al. (2014a)]**.

The sharing of an active ‘visual’ area both in sighted and blind participants across visual and tactile task modalities implies a more abstract, *supramodal* representation of specific information content. Supramodal brain regions may share a representation of the perceived stimuli independent of the input format from the sensory modality conveying the information to the brain (**Figure 2**).

As vision has long been considered crucial to explore and represent external sensory stimuli (that are processed along a segregated, but hierarchically organized, network of brain areas), supramodal responses were first assessed within the well-known visual functional pathways (e.g., Milner and Goodale, 1995; Goodale and Milner, 2006; Handjaras et al., 2012).

Supramodality has more recently been shown to be involved in integrated semantic representations and affective processing, ranging from action understanding to emotional and social functioning (Ricciardi et al., 2013, 2014a,b; Handjaras et al., 2015; Handjaras et al., 2016; Leo et al., 2016). Consequently, a more general ‘supramodal mechanism’ advances from simpler low-level to more complex sensory information toward more abstract, ‘conceptual’ representations.

## When Neuroscience ‘Touches’ Architecture: Do We Really Need Vision?

Therefore, according to this perspective, distinct elements of form and space in architectural perception may be processed and represented in highly specialized brain regions in a sensory modality-independent manner. In this sense, assessing the consistency or roughness of a material may recruit a supramodal neural content independently of the sense involved. The same may happen when exploring a complex object only by actively touching it. Rasmussen (1964) provided many examples which could be construed as supramodal architectural experiences *ante litteram*: he claimed, for instance, that just looking at the surface of a wall could evoke sensations of weightiness or lightness, hardness or softness.

On these premises, Mallgrave (2011) approached the *supramodal hypothesis* as a possible neural explanation of hapticity. As a matter of fact, by supporting the view of a more abstract nature of information representation, supramodality could theoretically comprehend and thus represent the neural correlate of hapticity and consequently provide the theoretical basis for its empirical investigation.

Nonetheless, if it is evident that vision is not solely responsible for spatial appraisal and perception as hapticity would imply, the notion of supramodality, in line with the intuition of a ‘sensory intensification’ in architectural appraisal (Van Kreij, 2008), further implies a more comprehensive overview on the embodiment of architectural experiences, shifting the balance beyond immediate sensory perception – not limited to a single sensory modality – toward higher cognitive, more abstract representations involving semantic, emotional and even social processing.

The conceptual potential of hapticity may have not been fully characterized yet, and therefore not fully exploited by architects. In addition, stating the predominance of the tactile sensory modality may be wrong. In fact, touch is constrained both spatially and temporally, as compared to vision. By definition, haptic perception happens in sequence, within a limited perceptual range and only through direct contact with the perceived object (Pons et al., 1987). In addition, the sense of touch relies more on specific properties, such as surface texture, than global ones, such as shape or localization in space (e.g., Lakatos and Marks, 1999; Podrebarac et al., 2014). On the other hand, vision relies on a parallel sensory processing, able to provide a comprehensive, *‘gestaltic’* perception over a distance and on a wider spatial extent (e.g., Gibson, 1979). Furthermore, functional neuroanatomy and psychophysiology demonstrated a perceptual and cognitive dominance of vision over other sensory modalities (Sereno et al., 1995; Gross, 1998).

Nonetheless, neuroscientists have recently referred to touch in a way that may take hapticity into account. From a phylogenetic perspective touch is an ‘earlier’ sense, developing prior to vision (even bacteria have it). Touch is a key element in communicating emotions and intimacy, maintaining and reinforcing social bonds (Suvilehto et al., 2015) and evidence shows that tactile stimulation accelerates brain development in infants (Guzzetta et al., 2009). Touch could even entail emotional involvement with inanimate objects (e.g., Hornik, 1992) and, from a functional perspective, it has been proven that the somatosensory cortices and the action recognition network show vicarious activations during non-visual socially relevant interactions (for a review: Keysers et al., 2010). Most importantly, haptic perception is crucial in determining a ‘sense of presence,’ which refers to the perception “of being immersed in the surrounding environment,” whereas vision often does not (Bracewell et al., 2008; Slater et al., 2009). As neuroscientists and architectural designers, we may ask ourselves whether environment appraisal indeed relies on such sensation of ‘being there’ (or ‘in touch,’ as it were) as the notion of hapticity seems to indicate, and to what extent it does so. Because the theorists of hapticity supported their idea of a multimodal sensing in the architectural experience by relying on the neuroscientific evidence that visual and non-visual information is equally processed and represented in the human brain, design decisions can truly integrate such knowledge to enhance architectural experience embracing the whole of the different sensory modalities. For instance, a recent study showed that symmetry is represented in the lateral occipital cortex in a supramodal fashion (Bauer et al., 2015) and many other design-relevant properties await to be investigated.

## Toward an Empirical *Responsibility Principle* in Architecture?

Since we spend the most part of our lives in buildings, our environment would greatly benefit from a perspective on architectural and urban design that is shared by both the architect and the neuroscientist. However, we must bear in mind that when dealing with the scientific method that characterizes life sciences, as suggested by Mallgrave (2015), architects must be prepared to address unexpected and possibly unwelcome empirical realities.

In fact, while the ‘neuro-turn’ has been welcomed by some architects as a way to “humanize” buildings (Pallasmaa, 2012) or to enhance architectural experience (Mallgrave, 2011), in other fields the same shift provoked an opposite reaction: some historians and sociologists see the fascination for neurosciences as a menace to human diversity and creativity (Fitzgerald and Callard, 2014), as a deeper knowledge of the molecular and neural correlates of human mind and behavior would prompt stereotyped approaches to design.

Many socially relevant research questions could be explored by neuroscience and architecture in synergy (see for instance: Pasqualini et al., 2013; Vartanian et al., 2013, 2015; Choo et al., 2016). Whereas currently the outcomes of this dialog and contamination between architecture and neuroscience are hardly predictable, we believe in the paramount importance of sharing knowledge among disciplines. Actually, the dialectics between the notions of hapticity and supramodality that we have described in this essay is a clear example of the weaknesses and potential strength of sharing theoretical models and terms. So, although hapticity suggests a primacy of touch that evidence from neuroscience does

not fully support, it also highlights the urge for a deeper understanding of processing or integration of multiple sensory modalities in environmental perception and appraisal. Actually, the comparison between these two different, but complementary approaches, may lead to novel observations regarding the people–environment relationships (e.g., concerning the architectural elements that may evoke the ‘sense of presence’), and even provide empirical foundations for a renewed evidence-based design theory (e.g., characterizing which visual and haptic cues evoke similar percepts or dissecting the role of each sensory modality in processing spatial information).

Such ambiguity of terms demands clarity. Many scientific fields that have matured toward the establishment of accepted methods had to come to terms with theoretical uncertainties such as those faced by architectural theorists and researchers right now. In scientific investigation, more accurate conceptual and linguistic choices should be made, in order to provide a common ground for the involved disciplines: specific terms must be preferred to fashionable and evocative ones, and evidence-based demonstrations should overcome speculations [Lilienfeld et al., 2015; see Franz (2005) as an example of such approach].

No infatuation for neuroscience will bring beneficial change to the architectural field if even eminent theorists still rely on verbal descriptions and speculations. On the contrary, if a paradigm shift awaits architecture, it cannot rely on a turn to neuroscience alone: architectural researchers now need to embody the *ethos* of empirical responsibility.

## Author Contributions

All authors listed, have made substantial, direct and intellectual contribution to the work, and approved it for publication.

## Conflict of Interest Statement

The authors declare that the research was conducted in the absence of any commercial or financial relationships that could be construed as a potential conflict of interest.
